# Transitioning Perspectives in Digital Health Through Phenomenology Integration

**DOI:** 10.2196/62691

**Published:** 2024-10-23

**Authors:** Maddalena Fiordelli

**Affiliations:** 1 Institute of Public Health, Faculty of Biomedical Sciences Università della Svizzera italiana Lugano Switzerland

**Keywords:** eHealth, digital health, phenomenology, phenomenological, participatory, health communication, health information, active listening, lived experience

## Abstract

The evolution of digital health, from its early days as eHealth to its current expansive scope, reflects a significant transformation in health care delivery and management. This transition underscores the integration of digital technologies across the health continuum from prevention and diagnosis to treatment and rehabilitation. The emergence of digital health has introduced innovative solutions but also posed challenges, particularly in aligning technological advancements with health needs, human experiences, and ethical considerations. This position paper aims to explore the integration of phenomenology in digital health, advocating for a paradigm that emphasizes the centrality of human experience in the design and implementation of digital health solutions. It specifically seeks to address challenges related to relevance for individuals who “speak” different languages, ensuring long-term use, addressing digital and health literacy, coordinating various sources, and navigating ethical issues in the rapidly evolving digital health landscape. Drawing upon years of research and practical experience in communication technologies and health, this paper uses a reflective approach to examine the intersection of digital health and phenomenology. It reviews the historical development of digital health, identifies the challenges faced during its evolution, and discusses the potential of phenomenological methods to enhance user-centered design and ethical practices in digital health. The integration of phenomenology into digital health facilitates a deeper understanding of user experiences, enabling the development of more responsive and ethical digital health solutions. Participatory design models, informed by phenomenological perspectives, offer a pathway to bridge the gap between technological innovation and human-centric health care. The paper highlights successful practices in digital health development, including mobile apps for vaccination decision-making and platforms for managing chronic conditions, illustrating the benefits of a phenomenological approach. Transitioning perspectives in digital health through phenomenology integration represents a critical step toward realizing the full potential of digital technologies in health care.

## The Promise of Digital Health

In 2001, this journal published an editorial listing the 10 characteristics, the 10 “e's” of eHealth, and providing a definition that was later extensively cited. The definition referred to eHealth as “an emerging field at the intersection of medical informatics, public health and business” [1]. But the multidisciplinary nature of eHealth was just a part of this visionary definition, which further underscored that “the term characterizes not only a technical development, but also a state-of-mind, a way of thinking, an attitude, and a commitment for networked, global thinking, to improve health care locally, regionally, and worldwide.” The potential and the promises encapsulated in this term were scrutinized for years to come. Today, the term seems far away, as it has been gradually replaced by the broader and more modern expression “digital health.” The latter better represents a complex reality by encompassing in the definition fast technological advancements together with social changes. Revisiting the 1970s reveals the emergence of various terminologies (eg, telematic and telehealth) [2], which have been stepwise encompassed in an interdisciplinary field and endeavor. According to World Health Organization (WHO) definition, “Digital health expands the concept of eHealth to include digital consumers” [3]; therefore, to reflect a part of the process, the end user can no longer be neglected but should become the core.

Digital health inherits from eHealth the disruptive potential of going beyond the technical development: it holds many promises. Digital health serves disparate purposes according to the health frameworks adopted, technological solutions used, foreseen aims, and contexts of adoption. If we consider the care continuum—from promotion to primary, secondary, and tertiary prevention, treatment, and recovery—digital health solutions exist for each stage, though with varying degrees of emphasis [4]. In terms of technological applications, we can enumerate many categories, such as geospatial and environmental, knowledge generators, imaging, personalized therapeutics, and so forth [5]. Digital health also started pervading not only different health settings, such as classical health care contexts like hospitals, specialized clinics, and private practice, but also citizens’ daily life and occupational environments (eg, well-being apps). Looking at the communication that digital health solutions facilitate or at the health communication aims they realize, we can enumerate the provision of information and motivational support, as well as patient engagement, facilitation of peer networking, and the possibility to meet individual patient needs in ways otherwise impossible [6].

## The Challenges Encountered by Digital Health

But beyond these promises, numerous challenges emerge, especially during the implementation phase of digital health solutions (Textbox 1). The discrepancy between market development and research outcomes is a primary challenge, already documented in systematic reviews 10 years ago [7]. The reality is that the market is pushing forward, but the consequence is that we often do not have solutions that are based on either theory or evidence where effectiveness remains unclear.

**Challenges to the promises of digital health.**

- Discrepancy between market development and research outcomes
- Quality and accessibility of information
- Health and digital literacy
- Multidisciplinary nature and communication challenges
- Integration into daily routines
- Coordination of stakeholders
- Sustainability
- Ethical concerns

Digital health develops following the evolution of information and communication technologies. This major change makes information, which is at the heart of health and health care, more accessible to everyone and increasingly present. However, as neologism like *infodemic* and recent scientific endeavor clearly showed [8], not all this information is of good quality, and this quality needs to be discerned at various levels.

Other crucial issues are related to the competence of the population, whether it be health literacy or digital health literacy [9]. Such issues are mostly investigated in specific subgroups of the population. We often discuss the digital divide among older adults in a simplistic way, viewing it primarily as a barrier to the adoption of digital health. However, current evidence is mixed, and older adults have demonstrated the ability to use technology effectively when the advantages and purposes are clear to them [10]. This example highlights that matters of literacy need to be framed in a broader and more complex context.

Digital health is multidisciplinary, and, in its development and implementation, it involves multiple audiences who speak different languages. In this context, health communication plays an important role as it has the potential to design proper strategies and to build the necessary common ground for reciprocal understanding [11]. Digital health solutions are attractive and innovative, but their adoption fails to endure over time [12,13] because it entails proper integration into daily habits, and this is a significant change that often gets unattended.

When promising digital health solutions have to be implemented, there are various audiences and sources that need to be coordinated, and the perspectives of these stakeholders must be taken into consideration [14]. If the intricacies of their power, legitimacy, and urgency are not considered, they might hinder the process [15]. Finally, 2 major chapters relate to the sustainability of technology and solutions on the one hand and ethical issues on the other. What often constitute a barrier to broad adoption of digital health solutions are indeed ethical matters [16] that might scare citizen and institutions: concerns about privacy, data governance, and so forth.

We could yet borrow the words of Gary Kreps [17] from a decade ago: “There is a long way to go for digital health information systems to reach their incredible potential.” If we compare the initial expectations about digital health, which were simplified perspectives that made us imagine easily achievable goals, with this more realistic outlook, some spontaneous questions arise: can we truly keep the initial promise? And if so, what are the concrete steps to do so?

## The Intersection of Digital Health and Phenomenology Through the Progressive Integration of Participatory Approaches

The chronological analysis of information and communication technologies and their development provides an additional perspective. Initially, the focus was almost exclusively on the content to be conveyed and its structure. When the web started its broader diffusion in the 90s, the approach to design was pretty much content based [18]. The web constituted an unprecedented opportunity of delivering information in different format, and the highest priority from the communication perspective was the one of the “few” senders back then, who decided how to frame and convey the message in the best way conceived. However, over the years, there has been a significant evolution, with increasing emphasis on the end user in the design process. It was about 10 years later that user-centered design approaches started to mushroom and eventually developed into in co-design, and later cocreation approaches [19].

This progression has led to the integration of participatory design models [20], highlighting the approach and sensitivity that are typical of the humanities and social sciences [21]. In addition to being participatory, this approach adopts a phenomenological perspective, valuing the experiences and voices of the user and even the patient. Wang and colleagues [22], in their umbrella review, outline a framework that facilitates the integration of digital experience at every stage of the development of digital health technologies and in all its iterations.

Phenomenology is the philosophical approach aimed at studying phenomena as they are experienced by individuals. Adopting a phenomenological approach therefore largely entails the incorporation of lived experience into the investigation [23]. This entails using the Husserlian lens of phenomenology [24], which underscores the necessity of *epochè* as an essential methodological approach for the examination of experiences. This is crucial in the development of conscious digital health solutions. By suspending preconceptions and assumptions, this methodology enables a critical examination and understanding of users’ lived experiences, thereby uncovering their genuine needs and challenge. This approach ensures that digital health solutions are authentically responsive to human contexts, fostering the development of empathetic, user-centered technologies that integrate seamlessly into daily life. Incorporating phenomenology into the development process facilitates the creation of innovative, ethically sound digital health tools that enhance the complexity of human experiences and lead to meaningful health outcomes.

The arguments presented in this paragraph illustrate how phenomenology could and should be integrated into the whole recurring cycle of design, development, testing, and implementation of a digital health solution. This contribution advocates for a paradigm that emphasizes the centrality of human experience in the design and implementation of digital health solutions [21]. In that, participatory design models constitute the approach adopted by successful practices in the digital health solution development and implementation cycle.

## Potential Pitfalls to the Integration

However, it is important to be aware that participatory approaches adoption for phenomenological integration can face various challenges. The first challenge concerns the complexity of identifying and involving all those who are directly or indirectly affected by the proposed technology. The direct and indirect beneficiaries or users of a digital health solution could be many, and their involvement is resource intensive. As it is for more classical research participation, involvement of the public into digital health solutions design, development, and implementation could face challenges in involving people [25,26]. It is essential to ensure the necessary resources to support participatory methods, which require a greater commitment in terms of time and reflection. This includes ensuring that those engaging with participatory methods hold the required sensitivity and competencies.

The second challenge addresses the need to balance the tension between the co-design phase as a separate entity and the existence of continuous and iterative co-design, as illustrated in the previous framework. So far, several attempts have been made throughout the process, but a systematic methodology has not yet been developed, possibly due to the inherently flexible nature of participatory methods [27]. Moreover, this approach is relatively recent and not yet widely shared. Acknowledging key methods, such as Q Methodology and phenomenological interviews [28-31] to name a few, which facilitate the integration of lived experiences, is a crucial first step toward systematizing this approach.

A third challenge is suggested by post phenomenological analysis and mediation theory [32], which demonstrate that technologies in an embodiment relation alter users by transforming their actions, motivations, and perceptions and making it impossible to revert to a pretechnological state without losing these changes. This highlights even further the importance of reflexivity in those who are collecting and analyzing the lived experiences of all those concerned with the digital health solution and, particularly, the end users [33].

Finally, there is the inherent tension between the 2 dimensions of digital health, namely, the health care sector and the technology sector. This tension manifests in terms of differences in implementation timelines and objectives and often complicates direct dialogue between the 2 spheres [5]. This poses many issues when developing and most of all implementing digital health, as it represents the root to the resistances from health care professionals [30]. In that, the listening and the integration of stakeholders’ perspectives remain though the only viable solution, as health care professional and technology developers’ lived experiences are essential to incorporate.

## The Empirical Demonstration of a Viable Option

The content of this work is informed by the practices I share in the following, which represent direct working experiences of the past 10 years of my research career. I have had the privilege of participating in various projects for the development, adaptation, and evaluation of digital health interventions. The chronological trajectory of my experiences also reflects the evolving sensitivity toward participatory design methods as a way of integrating phenomenology, highlighting and witnessing their maturation over time. These 3 case studies exemplify that.

The first practice concerns the development of a mobile app aimed at helping parents inform themselves and make decisions about their children’s vaccinations [34,35]. This application was conceived following a top-down approach, based on existing evidence and theories. However, during the evaluation phase, the use of mixed methods allowed us to capture the direct experiences of users. Within Wan and colleagues’ [22] design evaluation framework, we were able to address the challenge of long-term use through careful listening to end user experiences. Two different experiences are evidenced by 2 quotes (Textbox 2), representative of shared experiences that support or criticize the application, paving the way for a potential new iteration of the technological solution’s design. This case study illustrates a limited yet valuable investigation of lived experiences, the results of which can improve digital user experience autonomy [22].

**Figure 1:**
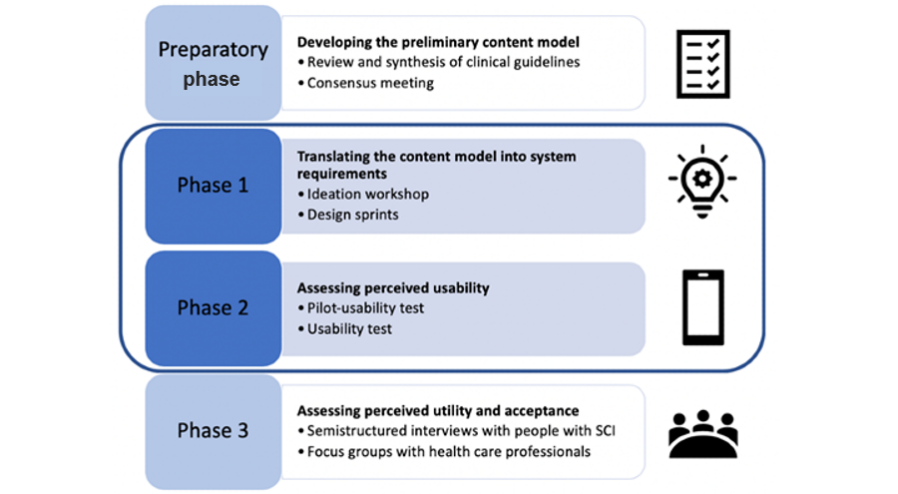
Co-design and development approach (reproduced from Amann et al [37], which is published under Creative Commons Attribution 4.0 International License [38]). SCI: spinal cord injury.

The second practice involves making the design phase participatory from the outset. For the creation of a prototype app for the prevention and self-management of pressure ulcers in patients with spinal cord injuries, we shared all decisions regarding content and functionality design with the various stakeholders involved: from health care professionals to individuals with spinal cord injuries. A comparison with the lived experiences of health care professionals allowed us to select scientific evidence where there was ambiguity or when the complexity of cases made it necessary [36]. Meanwhile, in ideation workshops, people with spinal cord injuries shared their significant experiences and the needs that emerged regarding the prevention of pressure ulcers [37]. This enabled researchers and developers to gain a deeper understanding of their reality, addressing challenges we have mentioned such as population literacy and the coordination of various sources. Before reaching the usability test phase, the development process was richly informed by multidisciplinary participatory methods (Figure 1) [37,38], from user experience design to health communication. These methods leveraged the collection of lived experiences to guide the decision-making process throughout all stages of the design, impacting personalization, information and content, navigation, and visualization [22].

**Figure 2:**
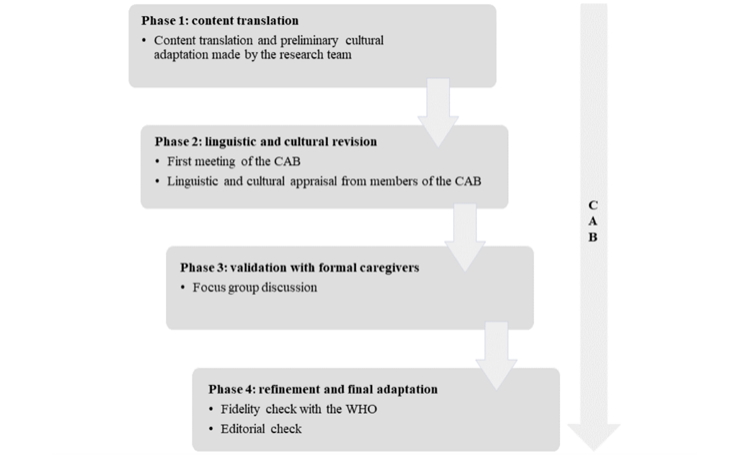
Flowchart of the adaptation process of iSupport in Switzerland (reproduced from Messina et al [40], which is published under Creative Commons Attribution 4.0 International License [38]). CAB: community advisory board; WHO: World Health Organization.

Adequately preparing a complex intervention for its implementation requires several research steps [39] that in our experience have taken more than 3 years to complete. This first involves adapting the content and delivery methods to the target population and culture, and second, testing its usability and effects to implement the program in the real-world context. This solution is *iSupport for dementia*, a support program for caregivers of people with dementia developed by a group of experts for the WHO in a generic version, which my colleagues and I have recently adapted, developed, and tested for the Swiss context in 3 national languages and in the form of a mobile app, desktop version, and paper manual. In this project, our goal was to advance from a less deep level of participation, such as that defined as “informing,” toward a more advanced form of collaboration and empowerment [25]. To achieve this objective, in addition to using mixed methods to include diverse experiences, we created a community advisory board [40], thus establishing a partnership that lasted throughout the project’s duration (Figure 2) [38,41]. Community advisory board members included caregivers, associations and government representatives, researchers, and technology developers. They were actively involved as industry experts throughout the entire research process, providing constant feedback on iSupport content and research strategies. This included participation in annual meetings, preliminary content review, support in selection, and active participation throughout the entire study. Thanks to this collaboration, which followed the entire project’s development, in accordance with the framework proposed by Wang et al [22], we were able to address challenges such as cultural and linguistic diversity and facilitate long-term adoption, developing a solution capable of meeting the needs of the involved audience. The multistakeholder participatory design served the purpose of refining both the design and the evaluation of the digital health solution. This integrated participatory approach has currently become a distinctive element of our public health research projects.

**Quotes from participants in the experiment of Morbiquiz.**

**Quote 1:**

- “Whether videos were present or not, it wouldn’t have made any difference. Because you could see this mother speaking, recounting her experience, but.... But I think that if there had been more videos with, let’s say, different opinions, from different mothers, it might have been more...more instructive, more of an overall picture....” (11225, both interviews [34])

**Quote 2:**

- “It’s a call to play, it’s a call to action. It’s so interesting to me, when you open the first question, I mean, now we have so many tools to navigate online and find the right answer, right? In fact, it invites you...to understand, read, analyze, correct? Then you give your answer. If it’s correct, great. You’re happy that what you saw was correct and you deepen your knowledge with the answer you receive. If it’s wrong, then you start to question the source you consulted, right? This challenge needs to be emphasized. It means getting involved, seeking information, and ultimately becoming active.” (11051, both interviews [34])

This contribution underscores the importance of investigating the lived experiences of all those involved into digital health solutions development and use as an effective tool for communication and persuasion in their design, development, and implementation. Favoring the listening of the different experiences emerges as a powerful means to establish empathy among the various stakeholders involved in the process. It highlights the need to value experiential knowledge to address and overcome common challenges in digital health, thus helping to fulfill the promises initially made. Furthermore, it emphasizes that this approach not only enhances social connections but also improves interactions in the context of developing solutions for digital health. The focus is on the importance of working on digital health with the many people involved, rather than simply working for them. This promotes a more participatory and user-centered approach, aiming to enhance collaboration and more effectively meet the needs of those involved in the development and use of digital health solutions. As this is accomplished, conducting further research to evaluate the effectiveness of integrating phenomenology into digital health solutions through participatory methods would significantly enhance the existing body of knowledge in the field.

## Conclusions

Transitioning perspectives in digital health through phenomenology integration represents a critical step toward realizing the full potential of digital technologies in health care. By centering the development and evaluation of digital health solutions on human experiences, we can overcome existing challenges and fulfill the promise of digital health to improve care outcomes and user engagement. Thanks to the sensitivity borrowed from the health communication perspective, this position paper calls for a collaborative effort among researchers, practitioners, and stakeholders to embrace phenomenological methods in digital health, ensuring that technological advancements enhance, rather than complicate, the health care experience.

## Abbreviations

WHO: World Health Organization

## References

[1] Eysenbach G (2001). What is e-health?. J Med Internet Res.

[2] Lovett JE, Bashshur RL (1979). Telemedicine in the USA. Telecommunications Policy.

[3] World Health Organization Digital health EURO.

[4] Cohen AB, Dorsey ER, Mathews SC, Bates DW, Safavi K (2020). A digital health industry cohort across the health continuum. NPJ Digit Med.

[5] Abernethy A, Adams L, Barrett M, Bechtel C, Brennan P, Butte A, Faulkner J, Fontaine E, Friedhoff S, Halamka J, Howell M, Johnson K, Long P, McGraw D, Miller R, Lee P, Perlin J, Rucker D, Sandy L, Savage L, Stump L, Tang P, Topol E, Tuckson R, Valdes K (2022). The promise of digital health: then, now, and the future. NAM Perspect.

[6] Robinson A, Oksuz U, Slight R, Slight S, Husband A (2020). Digital and mobile technologies to promote physical health behavior change and provide psychological support for patients undergoing elective surgery: meta-ethnography and systematic review. JMIR Mhealth Uhealth.

[7] Fiordelli M, Diviani N, Schulz PJ (2013). Mapping mHealth research: a decade of evolution. J Med Internet Res.

[8] Fiordelli M, Diviani N, Wac K, Wulfovich S (2022). Granting access to information is not enough: towards an integrated concept of health information acquisition. Quantifying Quality of Life: Incorporating Daily Life Into Medicine.

[9] Yang K, Hu Y, Qi H (2022). Digital health literacy: bibliometric analysis. J Med Internet Res.

[10] Bertolazzi A, Quaglia V, Bongelli R (2024). Barriers and facilitators to health technology adoption by older adults with chronic diseases: an integrative systematic review. BMC Public Health.

[11] Hu Y (2015). Health communication research in the digital age: a systematic review. J Commun Healthc.

[12] Mathews SC, McShea MJ, Hanley CL, Ravitz A, Labrique AB, Cohen AB (2019). Digital health: a path to validation. NPJ Digit Med.

[13] Zhu Y, Long Y, Wang H, Lee KP, Zhang L, Wang SJ (2024). Digital behavior change intervention designs for habit formation: systematic review. J Med Internet Res.

[14] Lampariello R, Ancellin-Panzani S (2021). Mastering stakeholders' engagement to reach national scale, sustainability and wide adoption of digital health initiatives: lessons learnt from Burkina Faso. Fam Med Community Health.

[15] Broekhuis M, Weering MD, Schuit C, Schürz S, van Velsen L (2021). Designing a stakeholder-inclusive service model for an eHealth service to support older adults in an active and social life. BMC Health Serv Res.

[16] Whitelaw S, Pellegrini DM, Mamas MA, Cowie M, van Spall HGC (2021). Barriers and facilitators of the uptake of digital health technology in cardiovascular care: a systematic scoping review. Eur Heart J Digit Health.

[17] Kreps GL (2014). Achieving the promise of digital health information systems. J Public Health Res.

[18] Garzotto F, Paolini P, Schwabe D (1993). HDM—a model-based approach to hypertext application design. ACM Trans Inf Syst.

[19] Kang X, Kang J, Chen W (2020). Conceptualization and research progress on web-based product co-design. Informatics.

[20] Nguyen AM, Rivera AM, Gualtieri L (2023). A new health care paradigm: the power of digital health and e-patients. Mayo Clin Proc Digit Health.

[21] Papoutsi C, Wherton J, Shaw S, Morrison C, Greenhalgh T (2021). Putting the social back into sociotechnical: case studies of co-design in digital health. J Am Med Inform Assoc.

[22] Wang T, Giunti G, Melles M, Goossens R (2022). Digital patient experience: umbrella systematic review. J Med Internet Res.

[23] Neubauer BE, Witkop CT, Varpio L (2019). How phenomenology can help us learn from the experiences of others. Perspect Med Educ.

[24] Tassone BG (2017). The relevance of Husserl’s phenomenological exploration of interiority to contemporary epistemology. Palgrave Commun.

[25] Vaughn LM, Jacquez F (2020). Participatory research methods—choice points in the research process. J Participatory Res Methods.

[26] O'Connor S, Hanlon P, O'Donnell CA, Garcia S, Glanville J, Mair FS (2016). Understanding factors affecting patient and public engagement and recruitment to digital health interventions: a systematic review of qualitative studies. BMC Med Inform Decis Mak.

[27] Messiha K, Chinapaw MJM, Ket HCFF, An Q, Anand-Kumar V, Longworth GR, Chastin S, Altenburg TM (2023). Systematic review of contemporary theories used for co-creation, co-design and co-production in public health. J Public Health (Oxf).

[28] Burton SV, Valenta AL, Starren J, Abraham J, Nelson T, Kochendorfer K, Hughes A, Harris B, Boyd A (2022). Examining perspectives on the adoption and use of computer-based patient-reported outcomes among clinicians and health professionals: a Q methodology study. J Am Med Inform Assoc.

[29] Callens C, Verhoest K, Klijn EH, Nõmmik S, Pina V, Brogaard L (2023). How Service Users Envision their Engagement in Processes of Collaborative Innovation: A Q-Methodological Study on User Involvement in eHealth Collaborations. Public Policy and Administration.

[30] Tomasella F, Morgan HM (2021). "Sometimes I don't have a pulse … and I'm still alive!" Interviews with healthcare professionals to explore their experiences of and views on population-based digital health technologies. Digit Health.

[31] Cummings M, Bradley J, Teal G (2022). Patient co-design of digital health storytelling tools for multimorbidity: a phenomenological study. Health Expect.

[32] Liberati N (2019). Emotions and Digital Technologies. HUMANA.MENTE Journal of Philosophical Studies.

[33] Finlay L (2002). "Outing" the researcher: the provenance, process, and practice of reflexivity. Qual Health Res.

[34] Fadda M, Galimberti E, Fiordelli M, Schulz PJ (2018). Evaluation of a mobile phone-based intervention to increase parents' knowledge about the measles-mumps-rubella vaccination and their psychological empowerment: mixed-method approach. JMIR Mhealth Uhealth.

[35] Fadda M, Galimberti E, Fiordelli M, Romanò L, Zanetti A, Schulz PJ (2017). Effectiveness of a smartphone app to increase parents' knowledge and empowerment in the MMR vaccination decision: a randomized controlled trial. Hum Vaccin Immunother.

[36] Fiordelli M, Zanini C, Amann J, Scheel-Sailer A, Brach M, Stucki G, Rubinelli S (2020). Selecting evidence-based content for inclusion in self-management apps for pressure injuries in individuals with spinal cord injury: participatory design study. JMIR Mhealth Uhealth.

[37] Amann J, Fiordelli M, Brach M, Bertschy S, Scheel-Sailer A, Rubinelli S (2020). Co-designing a self-management app prototype to support people with spinal cord injury in the prevention of pressure injuries: mixed methods study. JMIR Mhealth Uhealth.

[38] Attribution 4.0 International (CC BY 4.0). Creative Commons.

[39] Skivington K, Matthews L, Simpson SA, Craig P, Baird J, Blazeby JM, Boyd KA, Craig N, French DP, McIntosh E, Petticrew M, Rycroft-Malone J, White M, Moore L (2021). A new framework for developing and evaluating complex interventions: update of medical research council guidance. BMJ.

[40] Matthews AK, Anderson EE, Willis M, Castillo A, Choure W (2018). A community engagement advisory board as a strategy to improve research engagement and build institutional capacity for community-engaged research. J Clin Transl Sci.

[41] Messina A, Amati R, Annoni AM, Bano B, Albanese E, Fiordelli M (2024). Culturally adapting the world health organization digital intervention for family caregivers of people with dementia (iSupport): community-based participatory approach. JMIR Form Res.

